# Effect of Cavity Configuration (C Factor) on the Marginal Adaptation of Low-Shrinking Composite: A Comparative Ex Vivo Study

**DOI:** 10.1155/2011/159749

**Published:** 2011-09-19

**Authors:** Motaz A. Ghulman

**Affiliations:** Faculty of Dentistry, King Abdulaziz University, Jeddah, Saudi Arabia

## Abstract

*Aim*. To investigate the effect of C factor on marginal adaptation of low-shrinking composite (Silorane).The null hypothesis was that the marginal adaptation of “Silorane” is not affected by the cavity configuration. *Materials and Methods*. A Silorane based and a methacrylate based composites, with their corresponding self-etch adhesive systems “Filtek Silorane/Silorane Adhesive Bond System and Filtek Z250/Prompt L-Pop” respectively were used. Standardized cavities were prepared on the buccal surfaces of 100 maxillary premolars. Teeth were grouped into 5 groups (*n* = 20), for the 5 C factors. Restored teeth were subjected to thermocycling. Microleakage testing was done and linear dye penetration was assessed using a stereomicroscope. Statistical analysis was done using the Student's *t*-test. *Results*. For the methacrylate based systems' overall leakage score was significantly higher than the Silorane-based one (*P* = 0.034). For the methacrylate-based, leakage was found in all tested teeth groups except group 1 (C factor 1/5). For the Silorane, One-way ANOVA revealed a statistically significant increase in dye penetration in the 5th group (*P* = 0.010). *Conclusions*. The null hypothesis was rejected. The Silorane-based resin although it resulted in a statistically significant good marginal adaptation, it showed tendency toward a high leakage score with C-factor of 5.

## 1. Introduction 

 Polymerization shrinkage of resin composite restoratives remains a major impediment to their long-term clinical success. Contemporary composite materials shrink during polymerization, resulting in a volumetric reduction ranging from 1.5 to 5% depending on the molecular structure of the monomer, the amount of filler, and the rate of cure [1]. During polymerization, the volume of monomers is reduced, which creates sufficient shrinkage stresses to debond the material from dentin, thereby decreasing retention and increasing leakage.

 Factors that influence stress formation include volumetric polymerization shrinkage; elastic modulus and flow of the resin composite; adherence of the resin composite to the cavity walls; the configuration factor of the restoration. 

Cavity configuration factor (C-factor) is the ratio of the bonded surface area in a cavity to the unbonded surface area [2]. This means that, in a box-like class I cavity, there may be five times more bonded surface area than the unbonded surface area. 

 Much attention has been directed toward producing dentinal adhesives that withstand the forces involved during polymerization shrinkage of composite resins. Studies have shown that an effective dentin-adhesive bond depends on the wetting and penetration characteristics of the dentinal adhesive system and the reactivity of the treated dentinal surface. The structure of the collagen in the demineralized dentinal layer also seems to influence the behavior of the bond. Adhesive systems that do not excessively demineralize dentin, exposing the collagen fibers and leaving interwoven banded collagen in the demineralized layer, produce superior bond strengths [3]. 

 Lately, many research efforts are targeted to develop a nonshrinking high performance polymer for use as a matrix material for dental composite resins [4, 5]. One approach was the use of liquid crystalline monomers as a resin which was described to shrink less due to the transition of its nomadic phase to an isotropic amorphous state when photocured. Moszner et al. [6] published vinyl cyclopropane derivates as radical curing ring opening monomers, also suitable to copolymerize with common methacrylate-based resins. Eick and his collaborators [7], on the other hand, presented a different chemical approach focusing on the cationic ring opening spiro-ortho carbonates, especially in combination with epoxy monomers. 

 In the recent years, a new cationic ring opening monomer systems have been investigated with the target profile of a low-shrinking, highly reactive, and biocompatible composite that withstands the aggressive environment of the oral situation. 

The solution for this target profile was achieved by the development of siloranes. The name silorane derives from the combination of its chemical building blocks siloxanes and oxiranes [8]. It exhibits good mechanical properties comparable to those of clinically successful methacrylate-based composite materials [9] and low-shrinkage stress values in comparison to regular methacrylate composites [9–12]. Weinmann et al. [8] reported that the ring-opening chemistry of the Siloranes enables at the first time shrinkage values lower than 1 vol %.

Watts and Hindi[10] reported that the relatively slow shrinkage of Hermes, Silorane-based composite—possibly represents an advantage in relation to faster shrinking materials in that smaller and less extended marginal gaps may result from this “intrinsic soft start”. 

The low-shrinking composite is expected to be associated with better bonding and improved marginal sealability as it causes a more uniform stress distribution at the restorative composite-tooth interface. 

 In a study made by Gerdolle et al. in 2008 [13] comparing the sealability of a packable resin composite (Filtek P60), a compomer (Compoglass F), an Ormocer (Admira) and their associated bonding agents (Scotchbond 1, Excite, and Admira Bond, resp.), and a resin-modified glass ionomer (Fuji II LC), they failed to demonstrate the inverse relation between polymerization shrinkage and marginal sealing ability of various polymer restorative materials tested.

 In 2005 Palin et al. [14, 15] conducted series of studies based on the assumption that the novel low-shrink composites (RBCs) may offer a potential reduction in polymerization shrinkage and stresses. They tested the hydrolytic stability of OXI and SIL consisting of an oxirane/polyol and oxirane/siloxane resin-based matrix. They reported that the decreased water sorption, solubility and associated diffusion coefficient of the experimental silorane RBC, SIL may potentially improve hydrolytic stability of RBC restorations demonstrated by the nonsignificant decrease in biaxial flexure strength following medium-term immersion. 

 In 2008 Arisu et al. [16] studied the effect of occlusal loading on microleakage of class V cavities restored with a composite preceded by a two-step total-etch adhesive, a two-step self-etch adhesive, or a one-step self-etch adhesive. The two-step adhesive exhibited better marginal sealing than the all-in-one at the enamel margins under 250 N occlusal loading.

They stated that, surprisingly, Hermes (an experimental Silorane) in combination with its particularly designed adhesive “Hermes bond” showed leakage along the enamel margins—a result which was confirmed clinically in an ongoing clinical investigation. 

 Most recently in two different studies that tested the low-shrinking Siloranes' microshear and microtensile bond strength; lowest bond strength values were measured for silorane-based resin composite [17, 18]. Again, comparing Silorane with methacrylate-based composites revealed that, it was least affected by the change of C-factor [18].

## 2. Aim of study

 To compare between Silorane-based and methacrylate-based systems in marginal adaptation using different cavity configurations. The null hypothesis was that the marginal adaptation of a low-shrinking composite “Silorane” is not affected by the cavity configuration (C-factor).

## 3. Materials and Methods

### 3.1. Materials

#### 3.1.1. Adhesive/Composite Systems

 Low-shrinking silorane based and methacrylate-based Prompt L-Pop composite systems (Table 1) were experimented with.

#### 3.1.2. Teeth Specimens

 One hundred sound freshly extracted human maxillary premolar teeth were selected for the study. Teeth were extracted as a part of an orthodontic treatment plan. Selected teeth were free from caries, coronal fractures, or cracks. Teeth were debrided with hand scalers and cleaned with a rubber cup and slurry of pumice. They were then stored in saline solution at 4°C ready for the experiments.

### 3.2. Methods

#### 3.2.1. Specimen Preparation

 Standardized box-shaped cavities 2 × 2 × 2 mm were made on the teeth's buccal surfaces at their gingival halves. Cavities were positioned about one millimeter above the cementoenamel junction to ensure that the gingival cavity wall is in enamel. Positions and dimensions of buccal cavities were standardized through using a template (2 × 2 mm) prepared in a metal band strip.

Box cavities were made using no. 245 tungsten carbide burs in a high-speed handpiece under copious water spray. Depth of cavities was standardized by marking the burs at 2 mm length prior to use. A new bur was used after each ten prepared cavities. No bevels were added at any margin of the preparation. Cavity floors were inspected for absence of pulp exposures. Teeth were kept wet until the adhesive treatment procedure started.

#### 3.2.2. Specimen Grouping

Prepared teeth specimens were then classified randomly into 5 equal main groups of 20 teeth each (*n* = 20) relative to the number of cavity surface(s) to be bonded. In groups 1–4 the gingival wall was not allowed for bonding.

- Group 1: in which one cavity surface was allowed for bonding (C-factor = 1/5).
- Group 2: in which two cavity surfaces were allowed for bonding (C-factor = 2 /4).
- Group 3: in which three cavity surfaces were allowed for bonding (C-factor = 3/3).
- Group 4: in which four cavity surfaces were allowed for bonding (C-factor = 4/2).
- Group 5: in which all cavity surfaces (including the gingival one) were allowed for bonding (C-factor = 5/1).

#### 3.2.3. Cavities Restoration

For all of the above specimens the selected unbonded cavity wall(s) was premarked with a dot using a permanent colored marker on the corresponding surface and away from the cavity margin by about two mm for signaling as well as to facilitate identification.

Each of the five main groups was further subdivided into two subgroups A and B of ten specimens each (*n* = 10) for the two studied adhesive/composite systems, namely Adper Prompt L-Pop and Silorane, respectively. 

 Each of the two adhesives was applied to the preselected cavity walls to be bonded by following the manufacturers' instructions and under magnification using the Dental Operating Microscope (DOM). For the Adper Prompt L-Pop, the mixed adhesive was carefully applied and scrubbed on the preselected cavity wall(s) for fifteen seconds and gently dried for three to five seconds with compressed air. Bonding agent was then applied twice consecutively. During each application the material was rubbed for 15 seconds gently and thoroughly air-dried to remove the aqueous solvent. For the Silorane Adhesive Bond System, and the Silorane self-etch primer was applied to the pre-selected cavity wall(s) to be primed and bonded using the special minisponge applicator, rubbed gently for 15 seconds, then air-dispersed carefully. This was followed by light curing for 10 seconds. Silorane adhesive was then applied similarly and light-cured for 10 seconds also. 

 Box cavity samples of subgroups A and B were then restored incrementally with the particular resin-based composite, namely, Filtek Z250 and Filtek Silorane, respectively. Each increment was photo-irradiated (Heliolux DLX halogen light curing unit Vivadent, Schaan, Liechtenstein) for 40 seconds/1-mm increment. Restorations were then finished and polished with flexible discs (80−3 m, Soflex XT Pop-On, 3M ESPE, St. Paul, MN, USA, 3000 – 6000 rpm) under simultaneous water cooling. Finally restored teeth specimens were stored in water for 24 hours. 

 Restored teeth were subjected to thermocycling between 5°C and 55°C (5000 cycles dwelling time 30 seconds).

#### 3.2.4. Microleakage Testing

 Teeth specimens were covered with two layers of nail polish except for the restorations and approximately 1 mm margin around. The teeth were then dipped in a 2% methylene blue dye solution for 30 minutes (according to [20]). After dye penetration, the dye film on the tooth's surface was polished off with a 3M polishing disc (Soflex XT Pop-On 1982 SF). 

Each tooth was then sectioned vertically through the center of the restoration with a diamond disk at low speed under water coolant. The sectioned teeth were assessed using a stereomicroscope with an attached camera (Stereo-microscope 47507, Camera M 35, Zeiss, Göttingen, Germany) at × 24. 

#### 3.2.5. Image Analysis

**Figure 1:**
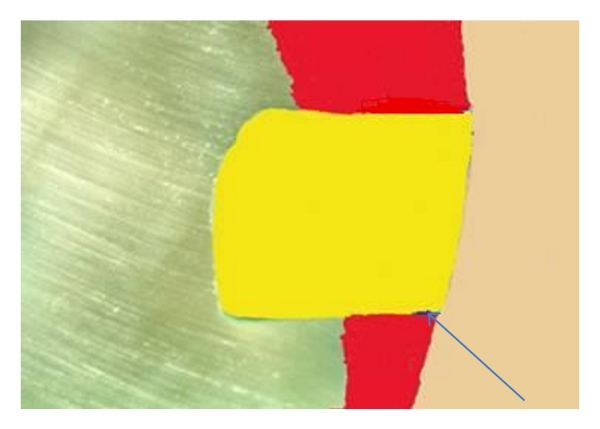
A sample photomicrograph of a tooth specimen showing color coding of tooth structure, filling material, and area of linear dye penetration (arrow).

**Figure 2:**
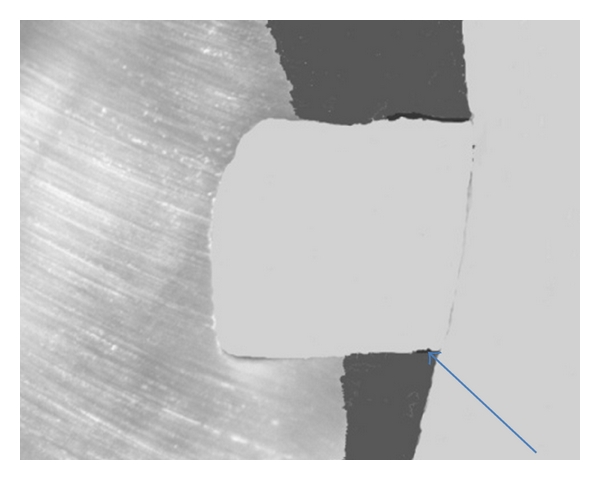
A photomicrograph of the same tooth specimen (Figure 1) showing conversion of color coded image into an 8-bit gray scale image. Arrow is pointing to the area of linear dye penetration.

Captured photomicrographs were transferred to a computer system for measurement of linear dye penetration at gingival margins using an image analysis software program (Image J 1.31b, USA). Processing of each photomicrograph was done before analysis to ensure standardization of each image for calculation. A color code was done for different structures of the image including tooth structure, filling material, and area of dye (Figure 1). Next, the colored image was converted into an 8-bit gray scale image (black and white) for easy selection of an appropriate threshold of a grey scale that ensures selection of the area of dye penetration only (Figure 2). On the 8-bit image, automated tracing of the area of interface was performed to select the desired area for calculation. A color code threshold of the area of dye penetration was undertaken prior to calculation. This was followed by automatic calculation of linear dye penetration at the gingival cavity margins.

#### 3.2.6. Statistical Analysis

Results were recorded, tabulated and statistically analyzed using one-way ANOVA. The test of Bonferroni was done as a post hoc test. Student s *t*-test was performed for Prompt-L-Pop and Silorane (SPSS. Version 12 for Windows).

## 4. Results

**Figure 3:**
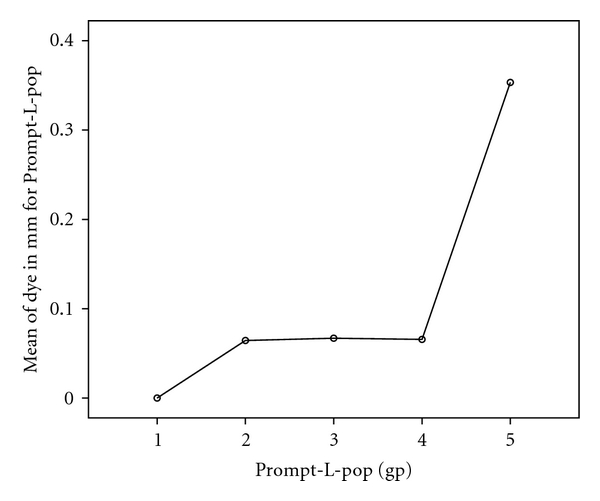
Result of Bonferroni test for the difference between cavity configuration groups for Prompt-L-Pop self-etch bonding agent.

**Figure 4:**
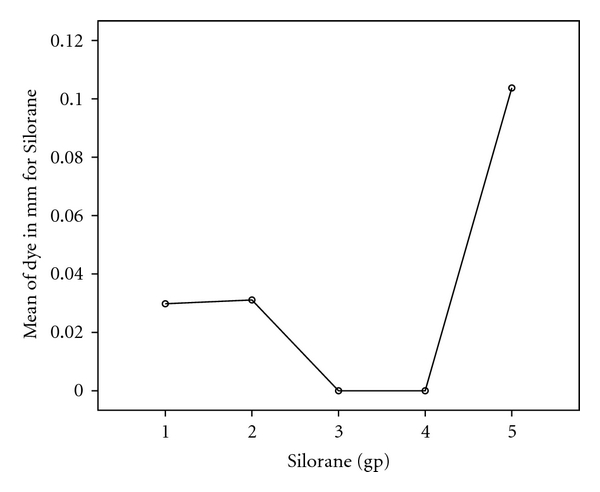
Result of Bonferroni test for the difference between cavity configuration groups for the Silorane adhesive bond.

One-way ANOVA for the results of dye penetration (in mm.) for Prompt-L-Pop and Silorane was presented in Tables 2 and 3. The test of Bonferroni's results was presented graphically in Figures 3 and 4. 

 For the Prompt-L-Pop, marginal leakage was detected in all tested teeth groups with exception of group 1 (C-factor 1/5). Thereafter, a minimum gradual increase in the mean linear dye penetration was perceived in groups 2, 3, and 4 (0.064 mm ±  .097, 0.066 mm ±  .150, and 0.067 mm ±  .145, resp.). A statistically significant marked increase in dye penetration was detected in the 5th group (x0.353 mm ±  .485) where the C-factor equals 5/1 (Table 2).  Figure 3 reflects the result of the Bonferroni test, a significant difference was found between group 5 and group 1 with the highest leakage score being in group 5. 

 For the Silorane groups, linear marginal leakage occurred in groups 1, 2 and maximized in group 5 with a mean of 0.030 mm ±  .067, 0.031 mm ±  .070, and 0.104 mm ±  .149, respectively. On the other hand, no leakage was detected in groups 3 and 4 (Table 3). One-way ANOVA revealed a statistically significant highest leakage score in group 5 (C-factor 5/1). Bonferroni test revealed a significant difference between group 5 and each of group 3 and 4 towards group 5 (Figure 4).

**Figure 5:**
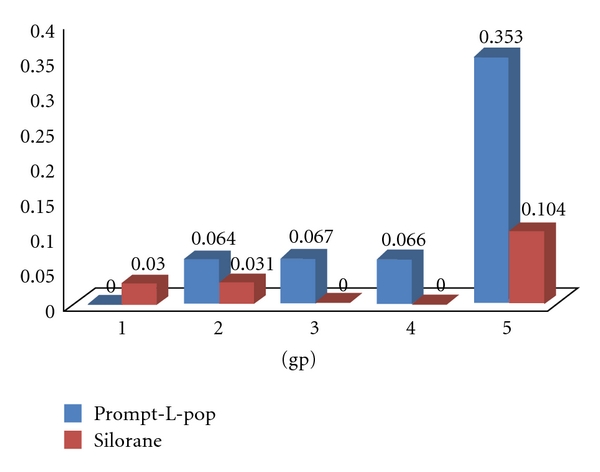
Mean dye penetration (in mm.) for Prompt-L-Pop and Silorane with different cavity configurations.

 Comparison between Silorane and Prompt-L-Pop in the overall mean leakage of the five groups collectively was made using paired samples statistics and paired samples correlation (Tables 4 and 5). The Prompt-L-Pop mean leakage score was found to be markedly higher than that of the Silorane (.1101 versus  .0329, resp.). This difference was found to be statistically significant (*P* =   0.034).  Figure 5 is a graphical representation of the mean dye penetration (in mm.) for Prompt-L-Pop and Silorane with different cavity configurations.

## 5. Discussion

 The present study was undertaken to investigate the effect of cavity configuration (C-factor) on marginal adaptation of low-shrinking Silorane-based composite (Filtek Silorane/Silorane Adhesive Bond System). A methacrylate-based composite: Filtek Z250/Prompt L-Pop was used as a base for comparison. Different cavity configurations (C-factors) were used to examine their possible effect on the linear leakage at the gingival margins of box-shaped cavities. Cavities on buccal surfaces of extracted human premolars were made with the gingival walls about 1mm coronal to the cemento-enamel junction to allow for an enamel and dentin gingival wall. 

 To facilitate careful application of each of the two adhesives only on the prespecified cavity wall(s) the procedure of adhesive application was done under high magnification (22x) using the Dental Operating Microscope (DOM). This was further made easier by the use of the minisponge applicator delivered with the Silorane Adhesive Bond System.

For the Adper Prompt L-Pop, two layers of the self-etch adhesive were applied with no waiting time for the second layer as recommended by the manufacturer for best performance of the material. The time of immersion of the specimens in 2% methylene blue dye for 30 minutes was selected according to the studies of Ernst et al. [19, 20]. They found it to be a suitable enough time for testing linear leakage at the class V cavities with good correlation to the Scanning Electron Microscopy for testing marginal adaptation at the enamel margins. 

 Results were presented as means and standard deviations. This latter parameter was found to be relatively high in both tested materials (Tables 2 and 3). The high standard deviation was related to the fact that in many of the specimens from the same group the leakage score was found to equal zero. In the Adper Prompt L-Pop subgroups, when the standard deviations were added, a clear general trend toward an increase in the leakage score with the increase in C-factor was found. Leakage in group 5 was found to be more than 5 times greater than that of groups 2, 3, and 4. Using the one-way ANOVA, this difference was found to be statistically significant. A possible explanation is that this is because in the above-mentioned group, the C factor was high (equals 5). This is in accordance with the explanation reported by Feilzer et al. [21, 22]. They described that a large unbonded area would facilitate composite plastic deformation during polymerization before the gel point is reached, thus reducing the final stress values. 

 It is known that the ratio of bonded versus unbonded areas (C factor) plays an important role with regard to the formation of shrinkage stress. The compensation of polymerization shrinkage by relaxation of the resin monomers is increasingly restricted by increasing C factor [23]. This explains the significantly higher leakage score related to groups 5 for both adhesives used in the present study.

 As for the Silorane, recorded linear gingival leakage for each tested group was significantly lower than that found with Prompt-L-Pop. Asmussen and Peutzfeldt [24] reported that the novel silorane-based resin composite had significantly lower polymerization contraction than the other methacrylate-based composites. This would appear to indicate that ring opening has taken place with a concomitant contraction that is relatively small. 

 Eick et al. [5] found that the stability and insolubility of siloranes in aqueous solutions containing epoxide hydrolase, porcine liver esterase, or dilute HCl enhances their potential as good candidate monomers for use in dental composite materials. The silorane resin is more hydrophobic than conventional methacrylate resins due to its siloxane backbone, so it results in reduced water uptake and related phenomena [7, 23].

Sauro et al. [25] detected micropermeability in several self-etching and etch-and-rinse adhesives with voids demonstrated along the resin-bonded interface except for Silorane and Optibond FL that showed an adhesive layer free from water trees and micro-permeability. The higher risk of defects at the resin-dentine interface, which may represent the pathway for hydrolytic and enzymatic degradation of resin-dentine bonds over time, would thus be minimized. 

On the other hand, no general trend relative to the C- factor was detected with the Silorane groups. It showed however the highest leakage score with a C factor of 5, although the extent of this linear leakage was only about one-third that of Prompt-L-Pop (0.104 (.149) and 0.353 (.485), resp.). The erratic behaviour of leakage scores of Silorane relative to C-factor denotes that C-factor might have an insignificant effect on Silorane. This is possibly related to the low-shrinking behaviour of Silorane [23]. 

The null hypothesis that the marginal adaptation of Silorane is not affected by the cavity configuration (C-factor) cannot be accepted. Although Silorane-based composite system was least affected by the change of C-factor and resulted in a statistically significant good marginal adaptation as compared to the methacrylate-based one, it showed tendency toward higher leakage score with C-factor of 5. This result was in concert with that of Klautau et al. [26]. 

Although both tested adhesives are self-etch, yet Silorane Adhesive Bond System is a two-step system while Prompt-L-Pop is a one-step all-in-one system. This may derive another explanation for the enhanced marginal adaptation that was proved by the lower linear leakage of the Silorane adhesive, based upon the aforementioned tactical difference in bonding. This explanation was in accordance with that of Sauro et al. [25] and Ola [17]. Nevertheless, it was reported in many previous studies that the two-step self-etch adhesives exhibit better marginal sealing than all-in-one [16, 27–29].

Ola [17] studied the microshear and microleakage behavior of Filtek Silorane/Silorane Adhesive Bond and Filtek Z250/Prompt L-Pop systems and reported that although both systems resulted in almost similar bond strength values, nanoleakage pathway and extent vary immensely among the different adhesives used suggesting a different behaviour of the adhesive joint in both materials during functioning and on aging with expected variation of bond durability and longevity of bonded restorations. 

## 6. Conclusions

Under the circumstances of the present investigation, the following conclusions can be drawn.

1. The null hypothesis that the marginal adaptation of a low-shrinking composite “Silorane” is not affected by the cavity configuration (C-factor) was rejected. However, Silorane was least affected by the change of C-factor
2. Marginal adaptation of Filtek Silorane/Silorane Adhesive Bond System as tested by linear dye penetration along the gingival wall of box cavities was found to be generally higher than the Filtek Z250/Prompt L-Pop system. This difference was found to be statistically significant.

## Figures and Tables

**Table 1:** Specification, constituent, and manufacturers for adhesives and composite resins used in the study.

Material	Specification	Constituent	Manufacturer
Filtek Silorane posterior restorative	Visible light activated silorane based restorative composite	(i) Silorane resin	3M ESPE Dental Product St. Paul, MN, USA
		(ii) Initiating system: camphorquinone, iodonium salt, electron donor	
		(iii) Fillers: Quartz filler	
		(iv) Yttrium fluoride with Average Particle Size (*μ*m) 0.47 Filler Load by Weight (%) 76%	
		(v) Stabilizers	
		(vi) Pigments	

Silorane Adhesive Bond System	Silorane Self-etch Primer:	Phosphorylated methacrylates	3M ESPE Dental Products St. Paul, MN, USA
		(i) Vitrebond copolymer	
		(ii) BisGMA	
		(iii) HEMA	
		(iv) Water	
		(v) Ethanol	
		(vi) Silane-treated silica filler	
		(vii) Initiators	
		(viii) Stabilizers	
	Silorane Adhesive Bond:	(i) Hydrophobic dimethacrylate	3M ESPE Dental Products St. Paul, MN, USA
		(ii) Phosphorylated methacrylates	
		(iii) TEGDMA	
		(iv) Silane-treated silica filler	
		(v) Initiators	
		(vi) Stabilizer	

Filtek Z250	Visible light activated methacrylate-based restorative composite	(i) The filler is zirconia/silica	3M ESPE Dental Products St. Paul, MN, USA
		(ii) The inorganic filler loading is 60% by volume “without silane treatment” with particle size range of 0.01 and 3.5 microns.	
		(iii) It contains Bis-GMA and TEGDMA resins.	

Adper Prompt L-Pop	Self-etch one-step bonding agent	Liquid 1 (red blister):	3M ESPE Dental Products St. Paul, MN, USA
		(i) Methacrylated phosphoric esters	
		(ii)Bis-GMA	
		(iii) Initiators based on camphorquinone	
		(iv) Stabilizers	
		Liquid 2 (yellow blister):	
		(i) Water	
		(ii) 2-Hydroxyethyl methacrylate (HEMA)	
		(iii) Polyalkenoic acid	
		(iv) Stabilizers	

**Table 2:** One-way ANOVA of the results of dye penetration (in mm.) for Prompt-L-Pop with different cavity configurations.

Prompt-L-Pop	*N*	Mean	Std. Deviation	Std. Error	*F*	*P*-value
gp1	10	0.000	0.000	0.000	3.758	0.010
gp2	10	0.064	0.091	0.029		
gp3	10	0.067	0.141	0.045		
gp4	10	0.066	0.138	0.044		
gp5	10	0.353	0.458	0.145		
Total	50	0.110	0.251	0.035		

**Table 3:** One-way ANOVA of the results of dye penetration (in mm.) for Silorane with different cavity configurations.

Silorane	*N*	Mean	Std. Deviation	Std. Error	*F*	*P*-value
gp1	10	0.030	0.063	0.020	3.219	0.021
gp2	10	0.031	0.066	0.021		
gp3	10	0.000	0.000	0.000		
gp4	10	0.000	0.000	0.000		
gp5	10	0.104	0.140	0.044		
Total	50	0.033	0.081	0.011		

**Table 4:** Paired samples statistics.

		Mean	*N*	Std. Deviation	Std. Error Mean
Pair 1	Prompt-L-Pop	.1101	50	.25345	.05069
	Silorane	.0329	50	.08207	.01641

**Table 5:** Paired samples correlations.

Bonding agent	*N*	Correlation	Sig.
Prompt-L-Pop & Silorane	100	0.424	0.034
